# How Women Use Digital Technologies for Health: Qualitative Interview and Focus Group Study

**DOI:** 10.2196/11481

**Published:** 2019-01-25

**Authors:** Deborah Lupton, Sarah Maslen

**Affiliations:** 1 News and Media Research Centre Faculty of Arts & Design University of Canberra Bruce Australia; 2 Faculty of Business, Government and Law University of Canberra Bruce Australia

**Keywords:** digital health, websites, online forums, social media, apps, wearable devices, women, Australia, feminist new materialism, qualitative research

## Abstract

**Background:**

A range of digital technologies are available to lay people to find, share, and generate health-related information. Few studies have directed attention specifically to how women are using these technologies from the diverse array available to them. Even fewer have focused on Australian women’s use of digital health.

**Objective:**

The Australian Women and Digital Health Project aimed to investigate which types of digital technologies women used regularly for health-related purposes and which they found most helpful and useful. Qualitative methods—semistructured interviews and focus groups—were employed to shed light on the situated complexities of the participants’ enactments of digital health technologies. The project adopted a feminist new materialism theoretical perspective, focusing on the affordances, relational connections, and affective forces that came together to open up or close off the agential capacities generated with and through these enactments.

**Methods:**

The project comprised two separate studies including a total of 66 women. In study 1, 36 women living in the city of Canberra took part in face-to-face interviews and focus groups, while study 2 involved telephone interviews with 30 women from other areas of Australia.

**Results:**

The affordances of search engines to locate health information and websites and social media platforms for providing information and peer support were highly used and valued. Affective forces such as the desire for trust, motivation, empowerment, reassurance, control, care, and connection emerged in the participants’ accounts. Agential capacities generated with and through digital health technologies included the capacity to seek and generate information and create a better sense of knowledge and expertise about bodies, illness, and health care, including the women’s own bodies and health, that of their families and friends, and that of their often anonymous online social networks. The participants referred time and again to appreciating the feelings of agency and control that using digital health technologies afforded them. When the technologies failed to work as expected, these agential capacities were not realized. Women responded with feelings of frustration, disappointment, and annoyance, leading them to become disenchanted with the possibilities of the digital technologies they had tried.

**Conclusions:**

The findings demonstrate the nuanced and complex ways in which the participants were engaging with and contributing to online sources of information and using these sources together with face-to-face encounters with doctors and other health care professionals and friends and family members. They highlight the lay forms of expertise that the women had developed in finding, assessing, and creating health knowledges. The study also emphasized the key role that many women play in providing advice and health care for family members not only as digitally engaged patients but also as digitally engaged carers.

## Introduction

Over the past three decades, a range of digital technologies for sharing and generating health and medical information has emerged. This began in the mid-1990s following the invention of the internet and the World Wide Web, with the establishment of information websites, email-enabled listservs, blogs, and online discussion forums for lay people to access details about health and medicine and share their own experiences. More recently, mobile devices constantly connected via Wi-Fi, social media platforms, mobile apps, and wearable self-tracking devices have become available to enable people to seek online information at any time and generate and share their own health data and experiences of health care and illness. Recent health care policy emphasizes the importance of active patient engagement with medical expertise, the incorporation of lay expertise into health care delivery, and patient responsibility for understanding and managing their chronic health conditions [1-4]. People are now often expected to be digitally engaged patients [5], actively using digital technologies to seek out information about health and medical issues and manage and promote their health.

The gendered dimensions of the use of digital health technologies have received little attention. Most of these technologies are designed for the universal user: an individual typically assumed to be white, male, and middle class. This can mean that devices do not fit well on female bodies or that menstrual tracking options are not initially included in smartwatch design (as was the case of the first Apple Watch, for example). Female bodily experiences such as menopause or problems with pelvic floor strength tend to be ignored [6-8]. Yet several studies have shown that women are higher users of online health and medical information than men [9,10]. Information websites, online discussion groups, and patient-authored blogs about health and medical topics have helped diverse groups of women, including those with metastatic breast cancer [11] or seeking information about breast cancer [12], women searching for information and support in relation to endometriosis [13], and women with multiple sclerosis [14]. The bulk of research related to women’s use of online health sources has focused on digital media for pregnancy and parenting [15]. This research has demonstrated how much women appreciate being able to easily find both medical expert advice and support from other women, who are often located across the globe, experiencing similar health and medical conditions, and willing to share information with each other.

Beyond the specific domains of pregnancy and early parenting, little research has focused on women’s use of apps, wearable devices, and social media for health-related purposes. Some studies involving both women and men have identified gendered differences in how people use health and fitness apps. For example, a French study [16] involving interviews with people aged from 20 to over 50 years who had tried using self-tracking diet and fitness apps noted that women were more likely to use diet apps, while more men used fitness apps. Older women were more resistant to using either type of app and more likely to use them for only a short time. Some survey-based research has shown that women tend to use health apps more than men, including studies in Hong Kong [17] and the United States [18]. However, a survey of older Germans (aged over 60 years) found that men were higher users of health apps than women [19], and another recent German survey of adults found little gender difference in health app use [20]. There is very little detailed qualitative research on how women use health and fitness apps. One study has investigated how adolescent girls interested in sports use fitness tracking apps, finding a degree of ambivalence in the participants, particularly in relation to their competitive elements [21].

Only a small number of studies published thus far have addressed Australian women’s use of digital health technologies. As with other geographical locations, most Australian-based research on women’s use of digital technologies for health-related purposes focuses on pregnancy and parenting. These studies have demonstrated that Australian women experiencing these life stages are keen users of social media and apps to find information, communicate and connect with other mothers, and track their pregnancy and children’s development [22-29]. A survey of Australian women who were pregnant or had a child under 3 years found that three-quarters had used a pregnancy app and half had used a parenting app [23]. Little research has investigated other groups of Australian women. One exception is a large survey of Australian young women (aged 18 to 24 years), which found that only 43% had used the internet to search for health information. Those women experiencing stigmatized conditions or symptoms (such as mental health problems) were more likely to have searched online than other participants [30]. Another study of young Australian women diagnosed with a sexually transmissible infection showed that they found both face-to-face and online sources valuable for advice and support [31].

In this paper, we present some key findings from the Australian Women and Digital Health Project. This project is innovative in several ways. First, it included Australian women across a range of ages, education levels, and geographic locations. Second, rather than focusing on specific digital health technologies, it covered the full range currently available to Australian women. Third, it investigated the contextual details of the participants’ lived experiences of digital health by using qualitative research methods that invited them to discuss these experiences in detail. Finally, concepts from feminist new materialism were employed to analyze the research materials. This theoretical perspective has not yet been adopted to any great extent in sociocultural analyses of digital health. We employed feminist new materialism to identify the affordances (of both technologies and human bodies), relational connections, and affective forces that came together to open up or close off the agential capacities generated with and through the participants’ enactments of digital health.

This approach generated many rich findings, providing novel insights into the women’s experiences of different types of digital health technologies. We focus in this paper on the findings that identify how women used health-related information websites, online discussion groups and social media, and apps and wearable devices.

## Methods

### Research Questions

The Australian Women and Digital Health Project aimed to investigate the following research questions: What digital technologies do women use regularly for health-related purposes, both for themselves and for any others (family members or friends)? Which do they find most and least helpful and useful? What kinds of digital health technologies would they like to see developed in the future? Qualitative methods—semistructured interviews and focus groups—were chosen because they are able to shed light on the situated complexities of the participants’ encounters with digital health technologies.

### Theoretical Perspective

The theoretical perspective adopted in this project is that of sociomaterialism and particularly feminist new materialism. While sociomaterialist perspectives acknowledge the importance of discourses, imaginaries, social relations, and interactions between people, they direct particular attention to the role played by nonhuman actors in humans’ enactments of technologies. This approach recognizes and emphasizes the relational engagements of people with technologies as well as with other people and the dynamic nature of these engagements. Humans and nonhumans (in this case, digital technologies) are viewed as working together to generate agential capacities, a term used in feminist new materialism theory to denote the ways in which people create action and meaning with and through things [32,33]. Empirical analysis is directed at identifying the ways in which “matter comes to matter” [32].

When this theoretical perspective is employed to analyze people’s experiences with digital health, it is acknowledged that human bodily sensations, digital technologies, and other humans (for example, family members, members of online communities, and medical practitioners) are involved in complex and ever-changing assemblages [34-36]. The affordances of both technologies and human bodies are brought together. Technological affordances include the uses that are designed into them, while human bodily affordances include enfleshed sensory responses and perceptions, thinking, and memory. The relational connections between actors in these assemblages, both human and nonhuman, and the affective forces generated with and through the intra-actions of these actors [37]—or how people feel emotionally in ways that compel action—contribute to the agential capacities that are created. When adopting a feminist new materialism approach, research materials such as interviews and focus group discussions are analyzed looking for the ways in which people describe their practices referring to these affordances, relational connections, and affective forces and how they work together to open up or close off agential capacities.

### Recruitment and Participants

The project comprised 2 separate studies. A total of 66 women participants across the 2 studies were involved in either interviews or focus groups about their use of digital health technologies.

Study 1 involved 3 sets of women living in Canberra, totaling 36 participants. The first set included a total of 11 women who attended an initial community forum advertised among women’s community health groups by the Women’s Centre for Health Matters, a community-based not-for-profit organization that works in Canberra and surrounding regions to improve women’s health. The participants who attended the forum were divided into 2 focus groups, one of which was led by the first author and the other by a staff member from the community center. Participant ages ranged from 28 to 65 years. The forum was used to identify key issues and to test and further develop the questions used for future interviews and focus groups.

Following this forum, another 12 participants (aged 21 to 63 years) were recruited to take part in individual face-to-face interviews. Three further focus groups with a total of 13 women were also conducted. Focus group 1 included 4 women with young children who were part of a support group for mothers living with mental health conditions (aged 25 to 30 years), focus group 2 comprised 6 women (aged 25 to 33 years) with young children, and focus group 3 included 3 women aged in their mid-to-late 50s. Of the total of 36 women involved across these Canberra participant groups, 28 identified their ancestry as Anglo-Celtic and 8 as Asian. Twenty-two participants reported university-level education, while 14 had high school or technical qualifications. These interviews and focus groups were conducted by two research assistants employed on the project. The participants were recruited using the Women’s Centre for Health Matter’s networks, personal contacts, advertising on relevant Facebook pages (such as those for mothers, people with disabilities, and women’s fitness and sporting groups in Canberra), and posters in public places around the city. The meetings took place in a range of locations, including places where the focus group participants usually met, homes, and cafes.

Study 2 involved telephone interviews with 30 women living in various locations around Australia. A market research company was commissioned to recruit the participants and conduct the interviews. Participant information and consent were provided online before the interviews were conducted. This group of participants was recruited using subquotas based on age to ensure a good spread of ages: 10 women aged 18 to 40 years, 10 women aged 41 to 60 years, and 10 women aged 61 years and over. Participants ranged in age from 22 to 74 years. Two-thirds lived in major cities; one-third lived in rural or remote Australia. Twenty participants lived in the state of New South Wales, 4 in Queensland, 5 in Victoria, and 1 in Western Australia. Twenty-five participants described themselves as having Anglo-Celtic ancestry, 1 as western European, 2 as southern European, 2 as Asian, and 1 as middle Eastern. Of this group, 14 reported university qualifications and the remaining 16 participants had high school or technical qualifications.

The same semistructured interview schedule was used with all participants. These questions provided the basis of the interviews and group discussions, but interviewers also probed participants for further comments and explanations of their responses. See Multimedia Appendix 1 for details on the questions asked in the interviews and focus groups.

### Ethics Approval and Consent

Ethics approval to conduct this research was granted by the University of Canberra’s human ethics research committee (reference number HREC 16-172). All participants were provided with project information and gave their consent to participate. They were all given pseudonyms to protect their anonymity.

### Analysis

All the group discussions were audio-taped and transcribed by a professional transcription company. The authors worked together to analyze the transcripts using inductive thematic analysis [38] informed by the feminist new materialism approaches outlined earlier. This involved identifying recurring themes within and across each group discussion by reading and rereading the transcripts, locating the places where the participants talked about the digital information that they accessed from online media, and considering the different ways in which human-technological assemblages came together in the participants’ accounts. We focused in particular on identifying the affordances, relational connections, affective forces, and agential capacities that were generated [35]. Our results are organized by topic (overview of information sources, information websites, discussion forums and social media, and health and fitness apps and wearable devices). Verbatim quotations from the discussions were chosen to provide support for the analysis.

## Results

### Overview of Health Information Sources

The interviews and focus group discussions opened with a contextualizing question asking participants what sources they currently used to access health information. They were specifically asked about every source listed in Table 1, which provides an overview of their responses; they also had the opportunity to list other sources. All of the participants said that they accessed both online sources and face-to-face sources of health information regularly. All referred to visiting doctors and other health care professionals, and the majority noted that in-person interactions with family and friends were also a key source of health information for them. For the most part, traditional media forms such as books were not highly used. However, printed pamphlets did remain influential sources for about half of the participants, particularly as they were available when the women were waiting at doctors’ offices for appointments. Other sources of health information were nominated by small numbers of participants (categorized as other in the table). These included a medical phone service, videos, podcasts, information sheet about a medication provided by pharmacist, asking a pharmacist for information, emailed newsletters from groups, magazines, newspapers, television advertisements, and television health programs/documentaries.

The next question asked participants to specify which digital health technologies they currently used. Again, they were specifically asked to respond to each technology listed in Table 2, which gives an overview of their responses.

As Table 2 demonstrates, using a search engine to search for health information online was a universal practice among the participants. Google Search was the only search engine mentioned by the participants: they typically referred to googling or consulting Dr Google when describing this practice. Health and fitness apps were used by over half of the participants. Social media were used less frequently (a third of participants), with Facebook groups most often mentioned as social media sources of information about health. One in five participants was currently using a wearable device for health-related purposes, with Fitbit (Fitbit Inc) fitness trackers and Apple Watches (Apple Inc) the most popular. Small numbers of women said they used digital self-care devices to manage a chronic health condition or exercise games like Wii Fit (Nintendo), while none reported using online physical fitness platforms like Strava. When asked if they used any other digital technology for health-related purposes that had not been listed, 2 said they played mind fitness games online, another 4 women referred to using email to send articles to family members or friends about health issues or being part of email groups set up for health-related topics, and 3 mentioned watching YouTube videos about health issues.

**Table 1:** Health information sources currently used by participants (N=66).

Source	Value, n (%)
Doctors/other health care providers	66 (100)
Online sources	66 (100)
Friends and family	56 (85)
Pamphlets	31 (48)
Books	12 (18)
Other	13 (20)

**Table 2:** Digital technologies currently used for health by participants (N=66).

Technology	Value, n (%)
Search engines	66 (100)
Websites	60 (90)
Apps	38 (57)
Social media	50 (33)
Online discussion forums	18 (27)
Wearable device	13 (20)
Exercise games	9 (13)
Self-care devices for chronic illnesses	9 (13)
Physical activity platforms	0 (0)
Other	9 (14)

### Health Information Websites

The majority of participants, regardless of their age, geographical location, or level of educational attainment, reported accessing health information websites. It was common for women to say that they went online very regularly: several times a week, in some cases. The definition of health-related information was quite broad in women’s accounts. It was interpreted by women to mean baby care; fitness advice; weight loss, diet, healthy eating, or cooking sites; discussion groups or social media groups, as well as sites that offered information about symptoms or medical conditions and treatments. Participants valued the currency of online information compared with traditional printed media, noting that websites were regularly updated.

Many women discussed going online to look for health information for a family member: a child (including adult children), grandchild, their partner, or elderly parent. One explained that she searches for information online on a weekly basis for family members’ health conditions.

I’ve got a son who has Asperger syndrome, which is a form of autism, and quite often I’ll access information to see if there’s anything new coming out. My husband has type 2 diabetes and a heart condition so quite often I’ll do research to see if there’s anything new coming out about that.

Susan, 56 years

Visits to medical practitioners were far less frequent. Susan, for instance, only visits her doctor quarterly on average. The women commented that consulting a doctor was much more time-consuming: it involved taking additional time to make an appointment that could fit into people’s schedules and to attend the appointment. They noted that they valued online sources highly because they were accessible at any time, free to use, and could be consulted at length, without time constraints. For some women, the expense of a doctor’s consultation was also a factor that deterred them from seeing doctors too often or too readily, if their doctor did not bulk-bill and there were out-of-pocket fees. People with poor access to the type of health services they needed also appreciated online resources. Those women who were caring for young children wanted to avoid the effort required to bring them along to an appointment.

The participants’ frequent recourse to websites for health information mostly did not diminish their trust or faith in medical expertise. When they were asked to nominate their major sources of health information, doctors and other health care professionals were mentioned by all the participants. Most women acknowledged that online sources supplemented rather than replaced the expertise and advice of medical practitioners. For example, one commented that she valued the more personal approach she obtains from a doctor’s visit. Doctors can examine her closely and draw on their experience in making a diagnosis. This expertise is not offered on the internet.

If you’re googling stuff, or you’re using the internet, or even social media for that matter, they don’t know the full story and they can’t see what you’re talking about—it’s just their opinion. But if you go to the doctor, the doctor has studied for this and they know what they’re looking for and they know how to deal with it.

Rachel, 38 years

The participants also described searching for further information once a diagnosis had been made by a medical professional. Online sources were used to fill gaps in information or explanations of illnesses that participants or their family members received from medical practitioners.

For instance, if you go to the doctor and they tell you you’ve got high blood pressure you would maybe just have a look to get more information about something that the doctor has diagnosed you with. Well with the doctor you’re only there for a limited amount of time so they probably can’t tell you every single little thing there is to do with it.

Jodie, 45 years

Although the participants were highly reliant on websites to find health information, they were not uncritical of the details they found there.

Google is most useful technology—I think it’s a great place to start, [but] when I do go on to Google I don’t go for the first one, I give a bit of time to research all the different sites to try and get a good overview. I don’t believe the first one that might pop up.

Audrey, 69 years

Participants were particularly concerned about the accuracy of websites funded by commercial interests. They outlined various strategies they used to determine whether they could trust information they found online. These strategies included looking for government-related websites (such as those run by departments of health), major health charities and organizations, and preferring Australian sources over non-Australian (because they were considered more relevant).

### Online Discussion Forums and Social Media

Many women also referred to the value of accessing peer communities such as patient support forums and Facebook groups established to share information about health and medical topics or specific conditions. The key benefits of these sources were the opportunity to share experiences as well as ask advice and find support from other people experiencing similar illnesses or life events. Online discussion forums and social media sites were described by women as providing support and advice from other people in their situation. They particularly valued being able to access a more personalized form of information that provided insights from like-minded others.

Just the really interesting things people put on there and real-life experiences and what they’ve gone through. They give you information, like links to go through, you can either take it or leave it. It’s up to you what you get out of it.

Houda, 45 years

Several women said that they appreciated the privacy that online forums and social media afforded them, meaning that they could see what other people were saying about health topics and contribute their experiences without needing to reveal their identity.

It’s more private, yeah, it’s definitely more... you know, you are able to look up safe without feeling embarrassed about if you were to talk to somebody about it. It’s not judgmental.

Lara, 33 years

Online forums and social media groups were sometimes used as a way of finding information based on lived experience that women were having difficulty accessing on medical websites or from their encounters with doctors. One gave an example of finding information for her adult daughter, who has Hashimoto disease (a disorder of the thyroid gland).

A lot of googling was discovering what this was and finding out there was a lot of people that had [this condition], and the things that made them sick. It wasn’t so much that we were talking on a forum but going in and finding what people had said. Not getting involved in that, but people were able to say, “Okay, I found out that this was blah blah blah and gluten really set me off.” And it was very good: very, very good.

Diane, 56 years

Other women recounted their experiences of contributing actively to an online forum or social media group. Facebook was the most commonly mentioned social media platform used by the participants for health and medical information and exchanging experiences. Some women, particularly those with young children or who were living with chronic health conditions, said that they were members of more than 10 Facebook groups related to health or parenting. A few women described using Instagram for following fitness influencers and Pinterest for healthy recipes and fitness tips.

For some women, online forums and social media were used in similar ways as health and medical websites: as a first source of information that helped them decide whether they needed to consult a doctor. Those women who used Facebook were sometimes members of numerous special-interest groups devoted to specific health topics, including diseases or medical conditions that they or their children had or nutrition or fitness groups. For example, a focus group participant said that she was caring for a child with allergies and had found an online forum for women in this situation to be a key source of advice and support.

For me, I have a forum that’s for mums with allergy kids. So I will often just go and read things on there because I find it really useful. I get lots of information that I wouldn’t have found otherwise. I do often actively ask questions, but questions that just like an experienced mother would know, not necessarily medical, yeah.

While the women were mostly very positive about discussion forums and social media they used for health information, advice, and support, some explained that participating in these peer-support communities could take an emotional toll. Some were also conscious of the potential limits of these platforms as an information source and so used them purposefully. A focus group participant who was part of several Facebook groups for mothers of young children described this tension.

I think it depends on what you’re looking for too, and what you’re asking because people have their own opinion and I kind of find they can borderline bully you on Facebook too... There’s a fine line between giving your opinion and then like pushing and pushing or being judgmental.

### Health and Fitness Apps and Wearable Devices

For those participants who used health and fitness apps, calorie-tracking apps such as MyFitnessPal and Weight Watchers and physical activity apps for monitoring heart rate, calories burned, and steps taken or apps providing workout or yoga programs and routines were by far the most often mentioned. Other apps nominated included those designed for the following purposes: medication reminders, self-diagnosis, medical insurance, first aid, Medicare (the universal state-funded health care system in Australia), water consumption, child vaccination, booking exercise classes, pelvic floor exercises, and sleep-tracking. Many of these apps were used for information purposes—to look up information about pharmaceuticals, for example, send out reminders, or to generate details about the users’ own bodies.

Several women who were using health and fitness self-tracking apps and wearable devices appreciated the better knowledge of their bodies that their practices gave them. They reported struggling with weight loss or attempting to increase their fitness levels. Being able to use an app or wearable device to closely monitor their bodies helped them exert control over their bodies. It is notable that women in their 50s, 60s, and even 70s reported using apps and wearable devices for these purposes. For example, one uses apps on her iPhone to help her monitor her weight-loss and exercise efforts.

I think it just helps you keep track of things a little bit better and easier. Before you had all these apps and things, you never knew how many steps you’d walked or never knew whether your heartbeat was fine or not unless you went to the doctor’s or the hospital. So this way it’s given you a little bit more control of what your body is telling you. Or knowledge I should say, not so much control, more knowledge about what your body is doing.

Pearl, 72 years

The women who used calorie and fitness tracking apps and wearable devices discussed how motivating they were, allowing them to set goals that they could strive to reach. Several women also referred to enjoying the notifications and reminders that apps and wearable devices sent them, again as a way of providing motivation to reaching goals. One woman uses MyFitnessPal to monitor her calorie intake and physical activity.

The app lets me know whether I’ve done well or whether I haven’t done well. Because you get a bit complacent with these sorts of things. And it sends me messages as well: “You haven’t logged on for a particular amount of time” or “You haven’t done enough steps today.” It just prompts you with bits of information like that. So it tracks how I’m going and it gives me reminders... Every time those little messages pop up, I think “Oh God, I haven’t done that yet!”

Julie, 51 years

Among the wearable devices for health and fitness mentioned by participants, Fitbit was the most highly used. Those women who used it described the benefits of being easily able to track their physical activity or their sleep patterns using the automated features of the device.

The Fitbit is easy. I don’t have to do anything, I’ll just wear it and set goals and that’s it. It’s easy and very measurable. It’s quantifiable, there’s no point in having goals if you can’t quantify it and you can’t measure it, well Fitbit makes both of those very easy. I wish I’d had it a lot further back. Even 10 years ago, this was a dream.

Robyn, 64 years

Apps to monitor reproductive health and parenting were also popular among the younger women. Several women used period tracking apps such as Clue to help them identify patterns in their menstrual cycle and generate notifications about when their next period could be expected. Women who were pregnant or had young children had used apps for tracking the progress of their pregnancy and finding information (apps such as Ovia and What to Expect When You’re Expecting), child vaccination records, infant development monitoring (in particular, the Wonder Weeks app), and parenting advice (for example, Baby Center). These apps served a combination of information provision and generating new data about the infants’ health and development. Some of the women in one of the focus groups said that they used apps to track the habits and routines of their infants. They had a conversation in which they talked about apps for tracking feeding, nappy changes, and sleep. They said that they found these apps helpful because “we’re still new mums,” as one woman put it, and “you’re just so tired all the time,” another added.

While app and wearable device use could be experienced as empowering and motivating, affective responses such as frustration, irritation, guilt, shame, and disappointment were expressed by some participants who had tried health and fitness apps. Chief among the complaints was poor design: the app did not work properly, kept crashing, or did not sync well to other devices. Some apps demanded too much time from users—this was particularly the case for calorie-counting apps that required users to input data manually each day. Other apps were considered not to be accurate enough. Some women began to find the constant reminders and notifications sent by apps or wearable devices to be overly intrusive. The women in the 2 focus groups comprised of new mothers, for example, noted that while they found self-tracking fitness apps and wearable devices to be helpful before having their babies, they no longer had the time or desire to use them. They commented on the guilt and shame that these technologies could incite in them and also the lack of acknowledgement by the devices or apps that their lives had changed so enormously. One participant observed this of her Garmin smartwatch.

I wish that there was a thing that during pregnancy where that I could put in and say “I'm pregnant,” because I got those notes that your sleep is really irregular, and I was like, “Because I’m pregnant!” ...It’s almost like it’s shaming you.

## Discussion

### Principal Findings

The findings from the Australian Women and Digital Health Project revealed new insights into how Australian women across a range of ages, geographical locations, and education levels are using the spectrum of digital technologies available to manage and support their health and well-being. There are no previous qualitative studies with which these findings can be directly compared, but they demonstrate that it is not only Australian women in the life stage of pregnancy or early parenting [22-29] who regularly seek information online and actively use social media and apps.

Older digital tools and sources such as search engines, websites, and online discussion forums are often neglected in contemporary discussions of the potential of digital health, while newer digital media such as apps, wearable devices, and social media receive high levels of attention. An important finding from our project is that the older digital media remain very highly used and valued among the participants. Regardless of their sociodemographic characteristics, our participants were avid users of online tools and resources such as search engines and websites to find health and medical information. They referred to valuing the affordances of instant and up-to-date information, the opportunity to search for information privately or anonymously, and the peer support that they could find online. Those women who used apps and wearable devices appreciated the opportunity to automatically monitor their bodies, engender motivation, and work toward health and fitness goals. The participants were actively working with digital technologies to source, assess, and apply health information and advice to their circumstances. They embraced the ideal of responsible citizenship promulgated by the digitally engaged patient discourse [5].

The participants’ accounts revealed that a range of agential capacities was generated with and through women’s engagement with digital health technologies. These included the capacity to seek and generate information and create a better sense of knowledge and expertise about bodies, illness, and health care, including the women’s own bodies and health, that of their families and friends, and that of their often anonymous online social networks. Affective forces such as motivation, reassurance, control, care, and connection emerged in the participants’ accounts. Women described a sense of empowerment from being able to readily access health information online and decide whether or not their concerns about their bodies or the health of family members were warranted, could be dealt with using lay remedies, or required a medical appointment. The participants referred time and again to the capacities of agency and control that using digital health technologies afforded them, including feeling as if they were able to better manage their own health, and in many cases, that of their family members. When the technologies failed to work as expected, these agential capacities were not realized. Women responded with feelings of frustration, disappointment, and annoyance, leading them to become disenchanted with the possibilities of the digital technologies they had tried.

Education levels or geographical location did not appear to play an important role in women’s use of digital health. However, age, life stage, and whether a participant was living with a chronic health condition or caring for a child or other family member with such a condition were influential. Reflecting general trends among Australians [39], the use of social media groups, apps, and wearable devices was more common among young and mid-life–aged women compared with those aged 65 years and over. Women with a chronic health condition or caring for a family member with such a condition, as well as those experiencing pregnancy or caring for young children, were among the most avid users of health websites, social media groups, and online forums, seeking peer support and alternative information sources to those offered by doctors as well as orthodox medical advice. However, these women could often be frustrated by the design of apps and wearable devices that did not recognize or cater for their needs in their current life stage or state of health. The capacities of these human- technology assemblages were closed down, as the technological affordances did not align well with their bodily affordances.

While a comparison with Australian men’s use of digital health cannot be made given that no previous research has focused on this group, it was notable that many participants reported frequently searching for information on behalf of their male partners as well as other family members. No women mentioned that any family member reciprocated this information sourcing on their behalf. Women have traditionally adopted this gendered role, taking responsibility for protecting and promoting the health of partners, children, and elderly parents [40,41]. These findings show that they are now using digital technologies to perform this type of reproductive citizenship [41] and family caring role.

Trust was an important affective force emerging in women’s accounts of how they evaluated online sources of information, what they did with this information, and how they interacted with their medical practitioners. The participants positioned their digital health activities as supplementing rather than replacing expert medical advice. As this suggests, online resources have increased the capacity of lay people to access both medical and lay expertise. Lay expertise was valued for its personalized insights into the everyday worlds of living with a condition and information about which treatments can work best, while medical expertise was valued for its authority, clinical experience, and facilitating access to other resources such as medical testing, drug prescriptions, and specialists. Medical experts tended to be positioned as being able to confirm and validate a self-diagnosis or self-sourced therapy, or alternatively, to allay fears that symptoms were serious. Rather than online health resources competing with expert health professionals, therefore, they were used in a complementary manner by the participants, often in ways that reduced their recourse to face-to-face medical services.

### Limitations

A limitation of the project is that while it included quite a diverse range of Australian women, there was an overrepresentation of women with a university education, those living in a metropolitan area, and those from Anglo-Celtic and English-speaking backgrounds. Further research should direct more attention to women who are members of more sociodemographically disadvantaged groups. Their experiences of digital health may be different, and their access to both digital technologies and health services tends to be more limited than women who are more advantaged [42]. In-depth studies focusing specifically on Australian men’s uses of digital health, another neglected area of research, would also draw further attention to the gendered nature of the enactment of digital health technologies.

### Conclusions

This research emphasized the situated dimensions of the range of digital technologies women used to support and promote their health and that of their family members as well as the nondigital elements of their health experiences and practices. The findings demonstrate that affordances, relational connections, and affective forces were important elements of the agential capacities that were opened up or closed off by women’s enactments of digital health. The research drew attention to the nuanced and complex ways in which the participants were engaging with and contributing to online sources of information and using these sources together with face-to-face encounters with doctors and other health care professionals and friends and family members. The findings highlight the lay forms of expertise that women have developed in finding, assessing, and creating health knowledges. The study also emphasizes the key role that many women play in providing advice and health care for family members. As this study demonstrated, many were actively using digital media to find information not just for themselves but for others. They were performing the roles of both digitally engaged patients [5] and digitally engaged carers.

## Appendix

Multimedia Appendix 1 Interview/focus group schedule.
