# Supplementary material for: How Women Use Digital Technologies for Health: Qualitative Interview and Focus Group Study
Source: J Med Internet Res. 2019 Jan 25;21(1):e11481. doi: 10.2196/11481 (PMC6367665; doi:10.2196/11481)
Supplement: Multimedia Appendix 1 [file jmir_v21i1e11481_app1.pdf]

AUSTRALIAN WOMEN AND DIGITAL HEALTH PROJECT

INTERVIEW AND FOCUS GROUP QUESTIONS

1. How do you tend to access health and medical information? (Prompts: Doctors or other healthcare providers, online sources, friends and family, books or pamphlets – any other sources?) Which of these do you find most useful or helpful for your needs? Please explain?
2. What digital technologies do you use for health and medicine at the moment? (Prompts: Online search engines to find health information, websites, apps, online discussion forums or social media groups, physical activity platforms like Strava, wearables like smartwatches or fitness trackers, exercise games like Wii Fit, self-care devices for chronic diseases – anything else?)
3. Have you signed up for the government's electronic patient record program (My Health Record)? Why or why not?
4. Do you use any of these technologies on behalf of family members (e.g. to find health information or track medical details for a partner, child, parent)?
5. Which of these technologies do you find most useful or helpful for your needs? What do you find helpful or useful about them – please explain?
6. Which haven't been useful? Please explain why? How do you think these technologies could be made more useful or helpful for you?
7. Do you ever think about where the personal information that these technologies collect about you or your loved ones (e.g. children) go and who uses it? Who do you think might access or use your information? Does this worry you at all?
8. If you could design the ideal digital health technology for your everyday needs, what would it be and what would it do?
9. Are there any other comments you would like to add about digital health technologies and how you use them?
